# Association between metabolic syndrome components and gingival bleeding is women-specific: a nested cross-sectional study

**DOI:** 10.1186/s12967-023-04072-z

**Published:** 2023-04-10

**Authors:** Davide Pietropaoli, Serena Altamura, Eleonora Ortu, Luca Guerrini, Theresa T. Pizarro, Claudio Ferri, Rita Del Pinto

**Affiliations:** 1 Department of Clinical Medicine, Public Health, Life and Environmental Sciences, University of L’Aquila, L’Aquila, Italy; 2 Center of Oral Diseases, Prevention and Translational Research-Dental Clinic, L’Aquila, Italy; 3 Oral Diseases and Systemic Interactions Study Group (ODISSY Group), L’Aquila, Italy; 4 Department of Medicine, Case Western Reserve University School of Medicine, Cleveland, OH 44106 USA; 5 Department of Pathology, Case Western Reserve University School of Medicine, Cleveland, OH 44106 USA; 6 Unit of Internal Medicine and Nephrology, Center for Hypertension and Cardiovascular Prevention, San Salvatore Hospital, L’Aquila, Italy

**Keywords:** Sex characteristics, Metabolic syndrome, Periodontal diseases, Smoking, Inflammation

## Abstract

**Background:**

Metabolic syndrome (MetS) is a cluster of atherosclerotic risk factors that increases cardiovascular risk. MetS has been associated with periodontitis, but the contribution of single MetS components and any possible sexual dimorphism in this relation remain undetermined.

**Methods:**

Using the third National Health and Nutrition Examination Survey (NHANES III), we performed a nested cross-sectional study to test whether individuals aged > 30 years undergoing periodontal evaluation (population) exposed to ≥ 1 MetS component (exposure) were at increased risk of bleeding/non-bleeding periodontal diseases (outcome) compared to nonexposed individuals, propensity score matched for sex, age, race/ethnicity, and income (controls). The association between MetS components combinations and periodontal diseases was explored overall and across subgroups by sex and smoking. Periodontal health status prediction based on MetS components was assessed.

**Results:**

In total, 2258 individuals (n. 1129/group) with nested clinical-demographic features were analyzed. Exposure was associated with gingival bleeding (+ 18% risk for every unitary increase in MetS components, and triple risk when all five were combined), but not with stable periodontitis; the association was specific for women, but not for men, irrespective of smoking. The only MetS feature with significant association in men was high BP with periodontitis. CRP levels significantly increased from health to disease only among exposed women. MetS components did not substantially improve the prediction of bleeding/non-bleeding periodontal disease.

**Conclusion:**

The observed women-specific association of gingival bleeding with single and combined MetS components advances gender and precision periodontology. Further research is needed to validate and expand these findings.

**Supplementary Information:**

The online version contains supplementary material available at 10.1186/s12967-023-04072-z.

## Background

Metabolic syndrome (MetS) is a multifactorial condition that increases the risk of atherosclerotic cardiovascular disease (CVD) and type 2 diabetes [1, 2]. Within the plurality of the diagnostic threshold applied, MetS is defined by the clustering of elevated arterial blood pressure (BP), high serum glucose and triglycerides, decreased high density lipoprotein cholesterol (HDL-c) levels, and increased waist circumference [3]. All MetS components contribute to increasing total cardiometabolic risk through specific major pathophysiological mechanisms, namely insulin resistance, visceral adiposity, atherogenic dyslipidemia, and endothelial dysfunction [4]. Additional contributing mechanisms have been implicated in the pathogenesis of MetS, including cell protein kinases and phosphatases alteration, suppression of the IRS1-IRS2 gene expression and function, lipid toxicity, alterations in the circadian rhythm, genetic and epigenetic factors, gut microbiome changes, and low-grade chronic inflammation [5]. Notably, emerging evidence convincingly demonstrates a sexual dimorphism in the epidemiology and physiopathology of MetS, with hormonal mechanisms influencing risk factors clustering and their consequences for metabolic and cardiovascular health [4].

Periodontitis is a chronic, progressively destructive, dysbiotic inflammatory disease of the tissues supporting the teeth with (epi)genetic and environmental risk factors and fluctuating disease course, characterized by gingival bleeding as a hallmark of active disease [6, 7]. With its milder forms affecting more than 50% of the global population, periodontitis is the sixth-most prevalent health condition worldwide and one of the top 10 more costly diseases globally [8, 9]. While the prevalence of periodontitis was reported to be higher in men, physiological hormonal fluctuations during life in women have been associated with changes in disease susceptibility [10, 11].

Several studies have so far investigated the relationships of periodontal diseases with diabetes, hypertension, obesity as defined by elevated body mass index (BMI) [12] and various definitions of MetS [13, 14], but none has addressed how the individual MetS factors contribute to the observed association, nor whether any possible sexual dimorphism exists in this relation. Visceral adiposity is indeed complementary and additive to BMI for its systemic impact [15]. Also, there is a knowledge gap as to how triglycerides and HDL-cholesterol relate with a condition with systemic implications as periodontitis in a sex-specific manner. Of note, the threshold value of single factors, like serum glucose and arterial BP, for the diagnosis of MetS is lower than what is required to define the corresponding pathologic condition, namely diabetes and hypertension. Thus, assessing whether the association persists when such alterations are defined according to MetS criteria warrants investigation for its potential impact in preventive medicine. In parallel, exploring any possible sex-specific feature in this relation would benefit gender and precision medicine.

The present study aimed to answer the clinical question whether adult individuals who were comparable in terms of potentially confounding factors (i.e., sex, age, race/ethnicity, and socioeconomic status) (population) exposed to at least one MetS component (exposure) were more likely to suffer from bleeding/non-bleeding periodontal diseases (outcome) than individuals not exposed to the same factors (controls), and whether the relation exhibited any sexual dimorphism. In addition, we adopted machine learning to assess the predictive power for periodontal diseases of MetS components in addition to traditional risk factors for the diseases, thereby contributing to the implementation of precision digital periodontology.

## Methods

### Study type

This is an analysis of cross-sectional data from The National Health and Nutrition Examination Survey (NHANES) III, conducted by the National Center for Health Statistics (NCHS) of the Centers for Disease Control and Prevention (CDC) between 1988 and 1994. NHANES III represents a stratified, multistage probability sample of the civilian non-institutionalized population in the 50 US states and the District of Columbia [16].

### Data source

NHANES III data can be viewed and accessed through the CDC– NCHS website. As an analysis of existing, anonymized data, the present study did not require Internal Review Board approval.

### Periodontal evaluation

The periodontal evaluation in NHANES III consisted of the assessment of periodontal probing depth (PPD), clinical attachment loss (CAL), and bleeding on probing (BoP) in each tooth in two sites (vestibular-mesial, vestibular-distal) in two quadrants, maxillary and mandibular, randomly chosen by a computerized function on the assumption that randomly selected sites would be representative of the global oral health status. The analysis of the third molars was excluded; therefore, it was possible to examine a maximum of 14 teeth and 28 sites per participant.

Participants were not eligible to periodontal evaluation if at least one of the following exclusion criteria was present: < 5 natural teeth, history of congenital heart disease, valvular heart disease, pacemaker or other artificial cardiac devices, bacterial endocarditis, rheumatic fever, kidney disease requiring dialysis, hemophilia, joint replacements, or malignancy; ongoing pregnancy and breastfeeding.

The extent of BoP was calculated as no. of bleeding sites/no. of probed sites (%). The dichotomous status of nonbleeding or bleeding gums was defined according to BoP (nonbleeding gums: BoP in < 10% of sites; bleeding gums: BoP in ≥ 10% of sites), Based on the American Academy of Periodontology/European Federation of Periodontology (AAP/EFP) consensus on the updated classification for periodontal and peri-implant diseases and conditions [17], stable periodontitis was defined as having BoP in < 10% of sites in the presence of clinical signs of periodontitis; unstable periodontitis was defined as having BoP in ≥ 10% of sites in the presence of clinical signs of periodontitis; gingivitis was defined as BoP in ≥ 10% of sites in individuals with intact periodontium; and healthy gums were defined as the absence of both periodontitis and bleeding (BoP < 10%).

In this study, only individuals aged 30 years and above were included, for consistency with previous literature [18, 19] and in agreement with the epidemiology of periodontitis as a disease of the adulthood and older age [20, 21].

### Definition of MetS components

According to the 2005 revision of the National Cholesterol Education Programme Adult Treatment Panel III (NCEP ATP III) guidelines [22], Mets components were defined as follows: (1) Fasting plasma glucose ≥ 100 mg/dL; (2) Arterial BP ≥ 130 mmHg (systolic component) or ≥ 85 mmHg (diastolic component); (3) Serum triglycerides ≥ 150 mg/dL; (4) Serum HDL-c < 40 mg/dL in men and < 50 mg/dL in women; (5) Waist circumference ≥ 102 cm in men and ≥ 88 cm in women. MetS was defined by the presence of at least 3 of the above listed factors. Individuals taking medications to lower their BP, their plasma glucose, or to improve their lipid profile were classified as suffering from hypertension, diabetes, or dyslipidemia, respectively, independent of their measured BP, plasma glucose, or serum lipid values. Plasma glucose was assessed in NHANES III using a modified hexokinase enzymatic method [23]. Arterial BP was measured in the sitting position with a mercury sphygmomanometer by trained and calibrated personnel according to a standardized protocol with three consecutive readings for each patient using the same arm; here, we used the average of these readings as provided by NHANES III. Serum triglycerides were measured enzymatically, and HDL-c was assessed following the precipitation of the other lipoproteins with a polyanion/divalent cation mixture [24].

### Classification of covariates

In addition to data indicated previously, other variables were used for descriptive or inferential purposes. In particular, participants were stratified by age categories (< 45, 45–64, > 65 years), sex, race (White, Black, other), income (poverty to income ratio [PIR], as defined by the U.S. Census Bureau), smoking habits (current, past, never), glycemic status (normoglycemia, pre-diabetes, or diabetes mellitus based on glycohemoglobin A1c [HbA1c] < 5.7%, 5.7–6.4%, or ≥ 6.5%, respectively), body mass index (BMI) (underweight, normal weight, overweight, or obese based on BMI < 18.5, 18.5–25, 25–30 or > 30, respectively). The Periodontal Inflamed Surface Area (PISA, mm^2^) was derived through the available periodontal indexes, as previously described [18]. Additional parameters included serum levels of inflammatory markers (C-reactive protein [CRP], ferritin, white blood cells [WBC]) and low-density lipoprotein cholesterol (LDL-c).

### Statistical analysis

To test our hypothesis in a controlled setting, we adopted a technique used by observational studies to improve effects estimates and infer cause-effects pathways [25]. Specifically, an exact 1:1 propensity score matching (PSM) for possible confounding factors in the association, namely sex, age, race/ethnicity, and income, was applied through a generalized logistic additive model [26]. The association of stable/unstable periodontitis and gingivitis with MetS and with all the possible combinations (n = 31) of single MetS factors was assessed in terms of odds ratios (OR; 95% confidence intervals, CI) by a multinomial adjusted logistic regression model with periodontal health status as the dependent variable. To avoid overadjustment given the balance achieved between the groups in terms of potential confounders, no further variables were added to the models. Stratified analyses based on sex and smoke were conducted. Interactions between exposure and smoking and the outcome was formally tested using relative excess risk due to interaction (RERI) and synergy index (SI) [27], and the CIs for the measures of additive interaction were estimated by applying the MOVER method [28]. A mediation analysis was performed to assess the possible mediating role of systemic inflammation (serum CRP) in the examined association [18].

In addition, three machine learning (ML) algorithms, namely stocastic gradient boosting modeling (GBM), extreme GB (XGB), and random forest (RF), were trained to test the ability to predict periodontal diseases based on MetS factors and systemic inflammation (serum CRP) on top of traditional risk factors for the diseases, namely age, smoking habits, and sex. The algorithms’ prediction ability before and after the addition of MetS components was tested through sensitivity and specificity analyses using the area under the receiving operating characteristic (ROC) curve (AUROC) [29]. ML algorithms were trained as previously reported [29].

Differences in clinical and demographic characteristics were assessed with the Wilcoxon test for continuous variables and with x^2^ test in case of categorical variables. Bonferroni correction was applied as appropriate. The data were analyzed as recorded, with no imputation for missing data. Statistical significance was set to p < 0.05. Statistical analyses were performed using R (v4.1.2).

## Results

Of the 31,311 total participants in NHANES III, 28% (n = 8614) received a complete periodontal evaluation. After applying the specified exclusion and inclusion criteria, a total of 7897 patients were eligible for PSM, and 2258 were 1:1 matched for the covariates of interest (Additional file 1: Fig. S1).

The exposed and unexposed groups (n. 1129/group) were exactly matched for sex (p = 1.000), age (p = 1.000), and race (p = 1.000) and did not differ in terms of income (p = 0.778), smoking habits (p = 0.152), and prevalent periodontitis (p = 0.064). Individuals exposed to at least one MetS component had higher BMI, glycated hemoglobin, systolic and diastolic BP, blood chemistry parameters, and CRP than unexposed; they also had more prevalent diabetes and hypertension than the comparison group (Table 1). Nearly one third of them (32.8%) fulfilled the criteria for MetS diagnosis. More than half of exposed individuals had high waist circumference (55.6%) and low HDL-c values (50.1%), while hypertriglyceridemia (38%), hypertension (36%), and fasting hyperglycemia (33.4%) affected more than 3 out of 10 patients in this group. Unexposed individuals were more often periodontally healthy (62.4%) compared to the counterpart. According to this, PISA, PPD and BoP were lower among non-exposed individuals, while CAL did not differ between groups (Table 1).

**Table 1:** Clinical and demographic characteristics of the analyzed sample

Level	Non-exposed	Exposed	p value
n	1129	1129	
Female (%)	574 (50.8)	574 (50.8)	1.000
Age (%)			
< 45 yr	783 (69.4)	783 (69.4)	1.000
45-65 yr	248 (22.0)	248 (22.0)	
> 65 yr	98 (8.7)	98 (8.7)	
Race (%)			
Black	319 (28.3)	319 (28.3)	1.000
Other	31 (2.7)	31 (2.7)	
White	779 (69.0)	779 (69.0)	
PIR (mean (SD))	2.86 (1.82)	2.84 (1.83)	0.778
Smokers (%)	561 (49.7)	596 (52.8)	0.152
BMI (%)			
Underweight	47 (4.2)	6 (0.5)	< 0.001
Normal	766 (67.9)	266 (23.6)	
Overweight	299 (26.5)	434 (38.4)	
Obese	16 (1.4)	423 (37.5)	
No. Of MetS factors (%)			
0	1129 (100.0)	0 (0.0)	< 0.001
1	0 (0.0)	441 (39.1)	
2	0 (0.0)	318 (28.2)	
3	0 (0.0)	206 (18.2)	
4	0 (0.0)	109 (9.7)	
5	0 (0.0)	55 (4.9)	
Increased WC (%)	0 (0.0)	628 (55.6)	< 0.001
High TG (%)	0 (0.0)	429 (38.0)	< 0.001
Low HDL (%)	0 (0.0)	566 (50.1)	< 0.001
High BP (%)	0 (0.0)	406 (36.0)	< 0.001
High glucose (%)	0 (0.0)	377 (33.4)	< 0.001
Periodontitis (%)	222 (19.7)	259 (22.9)	0.064
Periodontal status (%)			
Healthy	705 (62.4)	610 (54.0)	< 0.001
Gingivitis	202 (17.9)	260 (23.0)	
Stable periodontitis	143 (12.7)	137 (12.1)	
Active periodontitis	79 (7.0)	122 (10.8)	
PISA (mean (SD))	30.36 (61.21)	43.17 (79.81)	< 0.001
PPD (mean (SD))	2.35 (0.45)	2.43 (0.50)	< 0.001
CAL (mean (SD))	2.03 (0.91)	2.08 (0.90)	0.220
BoP (mean (SD))	7.44 (12.61)	10.13 (15.06)	< 0.001
Gingival bleeding (%)			
No BoP	596 (52.8)	517 (45.8)	< 0.001
BoP < 10%	252 (22.3)	230 (20.4)	
BoP ≥ 10%	281 (24.9)	382 (33.8)	
Diabetics (%)	0 (0.0)	74 (6.6)	< 0.001
Diabetics on insulin (%)	0 (0.0)	19 (28.8)	< 0.001
SBP (mean (SD))	115.53 (12.76)	124.29 (16.63)	< 0.001
DBP (mean (SD))	71.83 (8.14)	77.73 (10.25)	< 0.001
Hypertensives (%)	0 (0.0)	328 (29.2)	< 0.001
Hypertensives on HT pills (%)	0 (0.0)	158 (76.3)	< 0.001
BP ≥ 130/80 mmHg	280 (24.8)	570 (50.5)	< 0.001
BP ≥ 140/90 mmHg	55 (4.9)	239 (21.2)	< 0.001
HbA1c (%)			
Normal	1023 (91.0)	851 (75.7)	< 0.001
Pre-diabetes	99 (8.8)	188 (16.7)	
Diabetes	2 (0.2)	85 (7.6)	
WBC (mean (SD))	6.72 (2.01)	7.26 (2.20)	< 0.001
Lymphocytes (mean (SD))	2.16 (0.65)	2.35 (0.90)	< 0.001
Monocytes (mean (SD))	0.38 (0.18)	0.42 (0.21)	< 0.001
Granulocytes (mean (SD))	4.18 (1.65)	4.49 (1.77)	< 0.001
RBC (mean (SD))	4.62 (0.46)	4.73 (0.48)	< 0.001
Hb (mean (SD))	13.96 (1.41)	14.10 (1.53)	0.021
HTC (mean (SD))	41.42 (3.89)	41.76 (4.15)	0.047
PLT (mean (SD))	262.51 (63.12)	274.01 (72.93)	< 0.001
Ferritin (mean (SD))	113.32 (119.85)	145.10 (150.36)	< 0.001
LDL-c (mean (SD))	117.59 (32.28)	129.59 (33.99)	< 0.001
HDL-c (mean (SD))	60.81 (14.27)	47.27 (14.89)	< 0.001
CRP (mean (SD))	0.30 (0.43)	0.49 (0.99)	< 0.001
Glucose (mean (SD))	87.03 (7.18)	101.25 (35.55)	< 0.001
Creatinine (mean (SD))	1.06 (0.18)	1.07 (0.22)	0.068
Cholesterol (mean (SD))	199.25 (36.22)	210.57 (40.85)	< 0.001
Triglycerides (mean (SD))	80.67 (27.73)	153.61 (110.03)	< 0.001

Individuals in the exposed group have at least one MetS component, while non-exposed individuals have none

*BMI* Body Mass Index, *HbA1c* glycated hemoglobin, *PISA* periodontal inflamed surface area, *PPD* periodontal pocket depth, *CAL* clinical attachment loss, *BoP* bleeding on probing, *WBC* white blood cells, *RBC* red blood cells, *Hb* hemoglobin, *HCT* hematocrit, *PLT* platelets, *LDL-c* low density lipoprotein cholesterol, *HDL-c* high density lipoprotein cholesterol, *CRP* C-reactive protein, *SBP* systolic blood pressure, *DBP* diastolic blood pressure, *HT* hypertension

**Fig. 1:**
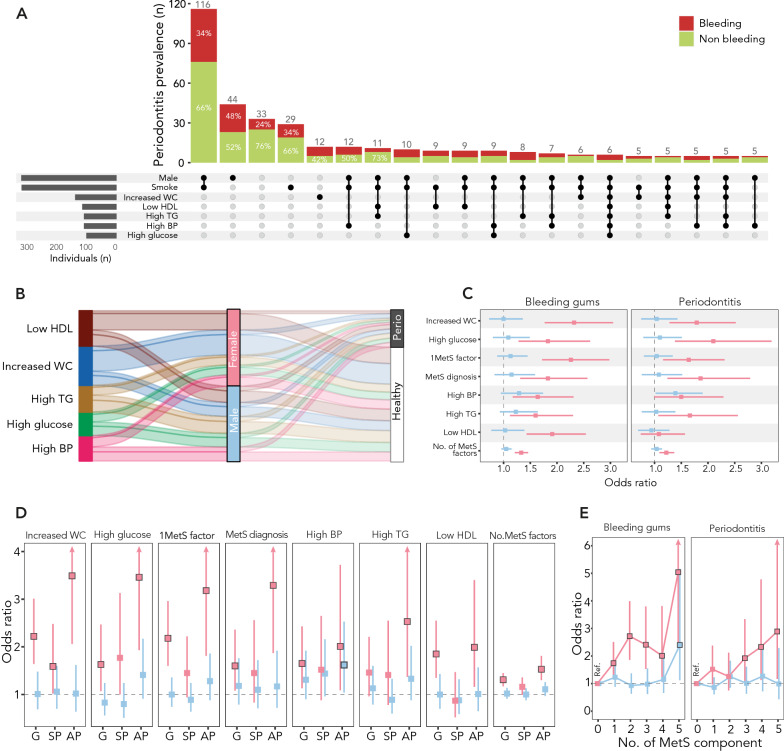
Sex-specific analysis of the relation between MetS components and periodontal health status. Panel **A** Combinations analysis (or multiple response analysis), reporting the prevalence of bleeding/non-bleeding periodontal diseases across combinations of sex, smoking habits, and MetS components (black dots and connection bars). Data visualization was limited to the twenty combinations with higher prevalence of periodontitis. Panel **B** Alluvial plot showing the distribution of MetS components (splines) across the reported axes strata (sex; periodontal condition). Each MetS component is indicated by a specific color. Panel **C** Association of bleeding/non-bleeding periodontal diseases with MetS definition, single MetS components, and any additional MetS component in women (pink) and men (blue). Panel **D** Association of MetS definition, single MetS components, and any additional MetS component with the periodontal disease spectrum (gingivitis; stable periodontitis; unstable periodontitis) by sex (women: pink; men: blue). Panel **E** Odds ratio (OR, 95% CI) of bleeding/non-bleeding periodontal diseases based on the number of MetS components in women (pink) and men (blue)

The sex-specific descriptive analysis showed a higher prevalence of periodontitis and smoking in men compared with women (Fig. 1A, Additional file 1: Table S1); exposed men were more often smokers compared to non-exposed men (65.4% vs 59.6%, p = 0.046), but no difference in prevalent periodontitis or gingival bleeding was found between exposed and non-exposed men (Additional file 1: Table S1). Conversely, no difference in smoking habits was found between exposed and non-exposed women (p = 0.863), and exposed women had higher prevalence of gingival bleeding and periodontitis than non-exposed women (Additional file 1: Table S1). While MetS factors were evenly distributed in men, high waist circumference and low HDL-c were those observed more frequently in women (Fig. 1B).

### Association between MetS components and gingival bleeding

First, we assessed the relation of MetS with gingival bleeding, a hallmark of gingivitis and unstable periodontitis.

Individuals exposed to MetS components had a 54% higher risk of gingival bleeding (i.e. either unstable periodontitis or gingivitis) (crude OR 1.54; 95% C.I. 1.29–1.85; p < 0.01) than nonexposed. All single MetS factors were associated with an increase in such risk. Specifically, the risk of gingival bleeding (unstable periodontitis or gingivitis) was 47% higher for elevated BP (OR 1.47; 95% C.I. 1.17–1.84; p = 0.001), 45% higher for hypertriglyceridemia (OR 1.45; 95% C.I. 1.16–1.81; p = 0.001), 44% higher for increased waist circumference (OR 1.44; 95% C.I. 1.19–1.76; p < 0.001), 41% higher for fasting hyperglycemia (OR 1.41; 95% C.I. 1.12–1.78; p = 0.004), and 37% higher for low HDL-c (OR 1.37; 95% C.I. 1.12–1.68; p = 0.002). In the presence of MetS, the risk of associated gingival bleeding was 44% higher (adjusted OR 1.44, 1.14–1.82, p = 0.002).

For every unitary increase in MetS components, there was a parallel increased risk of gingival bleeding by 18% (OR 1.18; 95% C.I. 1.10–1.25; p < 0.001). In addition, having at least two MetS components increased the risk of gingival bleeding by 55% compared to having none (OR 1.55; 95% C.I. 1.19–2.03; p = 0.001), and having all MetS factors increased the same risk by more than three times (OR 3.36; 95% C.I. 1.95–5.81; p < 0.001) (Additional file 1: Fig. S2).

### Association of MetS and its components with periodontal diseases spectrum

**Fig. 2:**
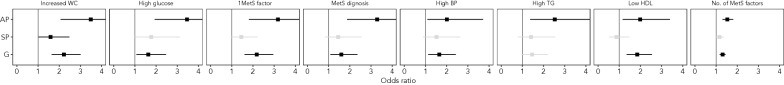
Forest plot of multinomial logistic regression for the risk of periodontal diseases spectrum based on MetS and its single components. Healthy periodontium was used as reference

We then examined the relationship of MetS with strata of periodontal diseases (stable periodontitis, gingivitis, unstable periodontitis), using healthy periodontium as reference. Multinomial logistic regression showed that MetS definition and any of its single components were associated with an increased risk of gingivitis or unstable periodontitis, but not with stable periodontitis, with the only exception of high BP (Fig. 2). Specifically, MetS definition was associated with 39% greater risk of gingivitis (OR 1.39; 95% C.I. 1.05–1.84; p = 0.021) and with 78% higher risk of unstable periodontitis (OR 1.78; 95% C.I. 1.24–2.56; p = 0.002) (Fig. 2). Each additional MetS component increased the risk of gingivitis by 16% (OR 1.16; 95% C.I. 1.07–1.25; p < 0.001) and that of unstable periodontitis by 26% (OR 1.26; 95% C.I. 1.14–1.39; p < 0.001) (Fig. 2). The greater association of MetS factors with unstable periodontitis was observed in the presence of hyperglycemia, which doubled the likelihood of this form of periodontal disease (OR 2.16; 95% C.I. 1.53–3.06; p < 0.001), followed by hypertriglyceridemia (OR 1.92; 95% C.I. 1.37–2.70; p < 0.001) and high BP (OR 1.87, 95% C.I. 1.31–2.66, p = 0.001). As to gingivitis, high BP was the single MetS factor best associated with the outcome (OR 1.50, 95% C.I. 1.14–1.96, p = 0.003), followed by increased waist circumference (OR 1.49, 95% C.I. 1.19–1.88, p = 0.001) and low HDL-c (OR 1.37, 95% C.I. 1.09–1.74, p = 0.008). Interestingly, high BP was also associated with an increased risk of stable periodontitis by 57% (OR 1.57; 95% C.I. 1.14–2.17; p = 0.006).

### Sex-specific association of MetS components with periodontal health status

We then assessed whether the association of MetS and its components with periodontal health status was sex-specific. Interestingly, we found that gingival bleeding was significantly associated with MetS definition, single MetS components, and any additional MetS component in women, but not in men (Fig. 1C). As for the sex-specific risk of periodontitis, high BP was the only feature with significant association with periodontitis in men (Fig. 1C, D). Low HDL-c was not associated with periodontitis independently of sex. High waist circumference in women was the single component with greater magnitude of association with bleeding/non-bleeding periodontal diseases. The risk of gingival bleeding based on the number of MetS components increased with their number in women, while it only became significant when all five were combined in men (Fig. 1E).

Given the observed sex-specific patterns of smoking habits and periodontal diseases prevalence, as described above (Additional file 1: Table S1), we assessed the RERI for smoking and exposure separately for men and women. No interaction was found between smoking and exposure, independently of sex (Additional file 1: Table S2). In men, smoke increased the risk of periodontal diseases irrespective of exposure; in women, smoking had no significant effect on the risk of periodontal diseases across strata of exposure.

In agreement with this, MetS definition, the number of MetS components, and the majority of single MetS factors were significantly associated with gingival bleeding among both smoker and non-smoker women (Table 2). The same association with periodontitis across smoking strata in women was only consistent for MetS definition, the number of MetS components, and the glucose criterion. Each additional MetS component increased the risk of gingival bleeding and that of periodontitis by 27% and 22%, respectively, in non-smoker women, and by 45% and 21%, respectively, in smoker women (Table 2). Conversely, no association was found between MetS features and bleeding/non-bleeding periodontal diseases across smoking strata in men (Table 2).

**Table 2:** Association of MetS components, their number, andMetS definition with periodontal health status across strata by sex and smoking habits

Smoke	Sex	Periodontal status	Variables	n	OR (95% C.I.)	p value
Smokers	Male	Periodontitis (ref. No periodontitis)	≥ 1MetS factor	694	1.06 (0.78–1.45)	0.717
			MetS diagnosis	694	1.05 (0.7–1.58)	0.815
			Increased WC	694	1.09 (0.74–1.6)	0.658
			High TG	694	0.91 (0.64–1.29)	0.607
			Low HDL	694	0.86 (0.59–1.24)	0.423
			High BP	694	1.62 (1.11–2.37)	0.012
			High glucose	694	1.13 (0.77–1.66)	0.545
			No. of MetS factors	694	1.04 (0.93–1.16)	0.53
		Bleeding gums (ref. No bleeding gums)	≥ 1MetS factor	694	1.2 (0.87–1.65)	0.271
			MetS diagnosis	694	1 (0.65–1.52)	0.987
			Increased WC	694	0.95 (0.64–1.41)	0.803
			High TG	694	1.14 (0.8–1.63)	0.454
			Low HDL	694	0.89 (0.61–1.3)	0.562
			High BP	694	1.44 (0.98–2.11)	0.067
			High glucose	694	1.06 (0.71–1.57)	0.778
			No. of MetS factors	694	1.04 (0.92–1.16)	0.54
	Female	Periodontitis (ref. No periodontitis)	≥ 1MetS factor	463	1.7 (1.03–2.83)	0.039
			MetS diagnosis	463	1.87 (1.03–3.41)	0.041
			Increased WC	463	1.6 (0.97–2.64)	0.068
			High TG	463	1.57 (0.85–2.93)	0.151
			Low HDL	463	1.31 (0.77–2.21)	0.316
			High BP	463	1.39 (0.73–2.65)	0.319
			High glucose	463	2.36 (1.24–4.48)	0.009
			No. of MetS factors	463	1.21 (1.03–1.44)	0.024
		Bleeding gums (ref. No bleeding gums)	≥ 1MetS factor	463	3.04 (1.87–4.93)	< 0.001
			MetS diagnosis	463	2.25 (1.3–3.88)	0.004
			Increased WC	463	2.88 (1.82–4.55)	< 0.001
			High TG	463	1.67 (0.95–2.94)	0.076
			Low HDL	463	2.84 (1.79–4.52)	< 0.001
			High BP	463	2.16 (1.23–3.79)	0.007
			High glucose	463	1.97 (1.07–3.63)	0.03
			No. of MetS factors	463	1.45 (1.24–1.69)	< 0.001
Non-smokers	Male	Periodontitis (ref. No periodontitis)	≥ 1MetS factor	416	0.82 (0.49–1.35)	0.434
			MetS diagnosis	416	1.09 (0.56–2.12)	0.793
			Increased WC	416	0.89 (0.47–1.68)	0.712
			High TG	416	1.04 (0.56–1.95)	0.901
			Low HDL	416	1.1 (0.61–1.97)	0.759
			High BP	416	1.03 (0.56–1.89)	0.928
			High glucose	416	1.02 (0.55–1.91)	0.948
			No. of MetS factors	416	1.01 (0.84–1.2)	0.947
		Bleeding gums (ref. No bleeding gums)	≥ 1MetS factor	416	1.05 (0.7–1.57)	0.801
			MetS diagnosis	416	1.46 (0.86–2.48)	0.166
			Increased WC	416	1.09 (0.66–1.79)	0.745
			High TG	416	1.51 (0.92–2.49)	0.101
			Low HDL	416	1.32 (0.82–2.12)	0.25
			High BP	416	1.08 (0.66–1.76)	0.762
			High glucose	416	1.14 (0.69–1.88)	0.608
			No. of MetS factors	416	1.09 (0.94–1.25)	0.254
	Female	Periodontitis (ref. No periodontitis)	≥ 1MetS factor	685	1.58 (0.99–2.53)	0.056
			MetS diagnosis	685	1.86 (1.07–3.24)	0.027
			Increased WC	685	2 (1.26–3.18)	0.003
			High TG	685	1.68 (0.91–3.09)	0.098
			Low HDL	685	0.84 (0.48–1.47)	0.543
			High BP	685	1.63 (0.93–2.85)	0.085
			High glucose	685	2.02 (1.16–3.52)	0.013
			No. of MetS factors	685	1.22 (1.04–1.43)	0.015
		Bleeding gums (ref. No bleeding gums)	≥ 1MetS factor	685	1.96 (1.4–2.76)	< 0.001
			MetS diagnosis	685	1.63 (1.06–2.51)	0.026
			Increased WC	685	2.07 (1.47–2.91)	< 0.001
			High TG	685	1.64 (1.02–2.63)	0.041
			Low HDL	685	1.56 (1.07–2.27)	0.019
			High BP	685	1.39 (0.9–2.14)	0.137
			High glucose	685	1.72 (1.11–2.67)	0.015
			No. of MetS factors	685	1.27 (1.12–1.43)	< 0.001

### Markers of systemic inflammation

**Fig. 3:**
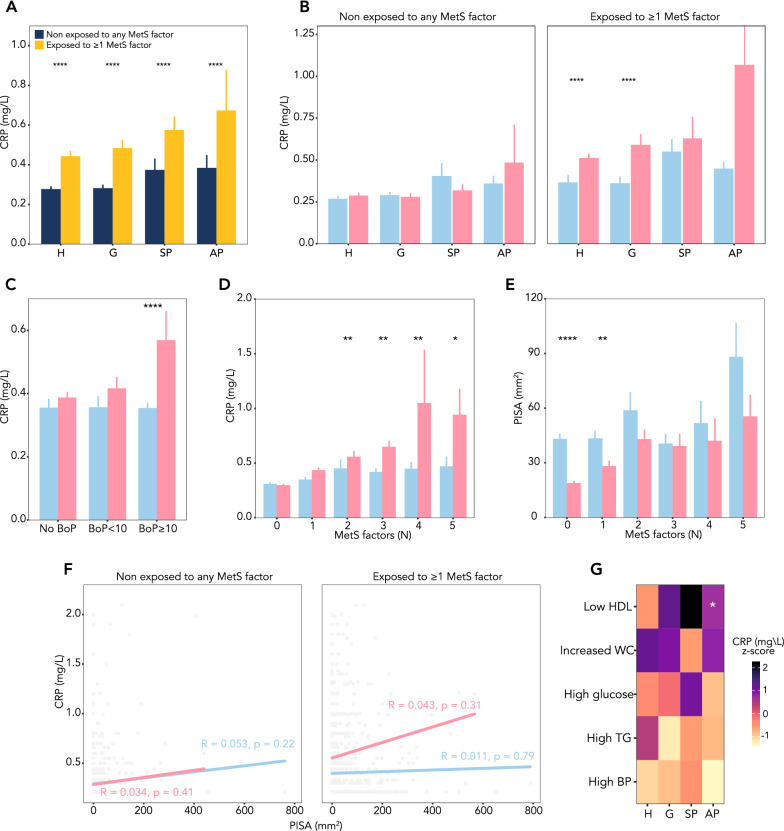
Systemic inflammation across periodontal health conditions during exposure to MetS factors follows a sex-specific pattern. Panel **A** Stratified analysis of serum CRP levels across periodontal health conditions based on exposure. Panel **B** Sex-stratified analysis of serum CRP levels of exposed and non-exposed across periodontal health conditions. Panel **C** Sex-stratified analysis of serum CRP levels by presence and extent of gingival bleeding. Panel **D** Sex-stratified analysis of serum CRP levels by incremental MetS components. Panel **E** Sex-stratified analysis of PISA by incremental MetS components. Panel **F** Sex-specific correlation of PISA and CRP levels based on exposure. Panel **G** Heatmap of CRP levels on individuals with single MetS components across periodontal health conditions. Asterisk indicates significant different mean CRP levels compared to healthy controls. Wilcoxon test adjusted for multiple comparisons with Bonferroni correction. Ns: not significant; *p < 0.05; **p < 0.01; p < 0.001; p < 0.0001

In exposed individuals, serum CRP levels increased progressively from healthy periodontium to gingivitis, stable periodontitis, and unstable periodontitis and were significantly higher than in non-exposed participants (Fig. 3A), reflecting elevated CRP levels in women across these conditions (Fig. 3B). Exposed women had higher CRP levels than exposed men both in the presence of a healthy periodontium and during gingivitis, a finding that was not detected among non-exposed individuals (Fig. 3B). Similarly, generalized gingival bleeding (BoP ≥ 10% of sites) was associated with significantly higher serum CRP levels than non-bleeding gums or localized bleeding (BoP < 10% of sites), a finding that was driven by higher CRP levels in women as compared to men (Fig. 3C). According to the mediation analysis, CRP mediated 24% of the observed association between periodontal health status and MetS components (Additional file 1: Fig. S3).

Interestingly, serum CRP levels increased with the number of MetS components in women, but not in men (Fig. 3D). The PISA also increased with the number of MetS factors (Fig. 3E), with men showing a global trend towards higher PISA as compared with women. PISA and CRP levels were not correlated based on exposure; similarly, they were not correlated in men with or without criteria for MetS definition (Fig. 3F). Interestingly, however, a linear correlation was found between PISA and CRP in women without criteria for MetS definition (Fig. 3F).

CRP levels were comparable across combinations of periodontal health conditions and MetS components, with the only exception of the finding of significantly higher CRP levels in individuals with unstable periodontitis and concomitant low HDL-c compared with periodontally healthy individuals (Fig. 3G).

### Prediction of periodontal health status based on MetS components

**Fig. 4:**
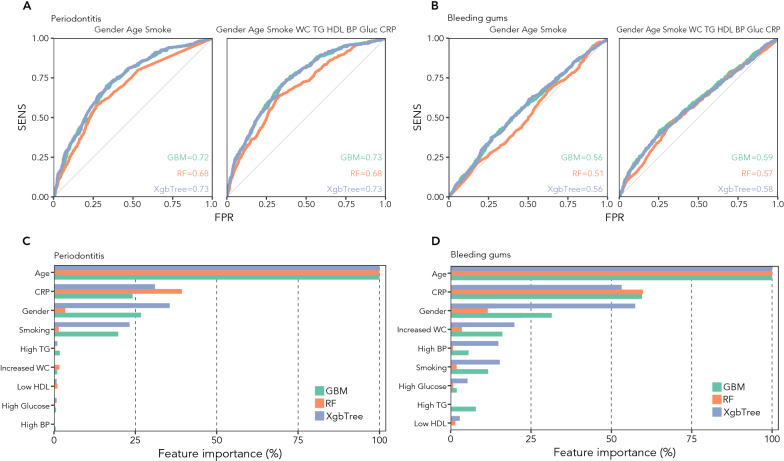
MetS components and systemic inflammation improve the prediction of gingival bleeding, but not that of periodontitis. Panel **A** AUROC curves showing the predictive power of GBM, RF, and XBG to discriminate periodontitis using only sex, age, and smoking habits (left), or with the addition of MetS factors and CRP (right). Panel **B** AUROC curves showing the predictive power of GBM, RF, and XGB to discriminate gingival bleeding using only sex, age, and smoking habits (left), or with the addition of MetS factors and CRP (right). Panels **C**, **D**. Bar graph of the relative importance of individual variables in the learning process of algorithms to identify periodontitis (panel **C**) and gingival bleeding (panel **D**)

Based on sex, age, and smoking habits, the ML algorithms could fairly predict periodontitis (AUROC GBM: 0.72; AUROC RF: 0.68; AUROC XGB: 0.73; Fig. 4A), but not gingival bleeding (AUROC GBM: 0.55; AUROC RF: 0.52; AUROC XGB: 0.54; Fig. 4B). Adding information about exposure and systemic inflammation did not substantially improve the ability of the models to predict gingival bleeding (AUROC GBM: 0.59; AUROC RF: 0.57; AUROC XGB: 0.59; Fig. 4B) or periodontitis (AUROC GBM: 0.73; AUROC RF: 0.68; AUROC XGB: 0.73; Fig. 4A). The ML performance did not improve using a different threshold for BoP, i.e. 0% and 30% (data not shown).

In terms of relative importance of variables to instruct the algorithms for periodontitis prediction, the most significant contribution to learning came from age, CRP, and sex, followed by smoking, while the MetS components did not substantially contribute to prediction (Fig. 4C). As for gingival bleeding, the same first three variables were followed for importance by high waist circumference, low HDL-c, and high BP, while smoking, high triglycerides, and high glucose provided progressively lower contribution to prediction (Fig. 4D).

## Discussion

Our findings indicate that MetS components are associated with gingival bleeding, a hallmark of gingivitis and unstable periodontitis, but not with stable periodontitis, and that the association is specific for women, but not for men, irrespective of smoking habits. The only MetS feature with significant association in men was high BP with periodontitis.

Women with at least one MetS component had higher systemic inflammation compared with men across the spectrum of periodontal health conditions, with increasing CRP levels from health to disease, a finding that was not seen in the absence of exposure to MetS factors. While no correlation was found between the local extent of inflammation and CRP levels in men, irrespective of exposure, the two were linearly correlated in women if the criteria for MetS definition were not met. However, MetS components were not relevant for the prediction of bleeding/non-bleeding periodontal disease beyond the resolution provided by age, sex, systemic inflammation, and smoking.

These data align with recent literature on the systemic impact of gingival bleeding and add novel information on the sex-specific relation between MetS and its single components with active periodontal diseases. In fact, very few studies so far conducted [14, 30] have examined the association of MetS with gingival bleeding or with the definition of periodontal disease activity according to AAP/EFP guidelines [31, 32], which represents indeed a crucial aspect to assess, given the better relation of gingival bleeding with systemic inflammation as compared with non-bleeding [33], independently of socioeconomic confounders and even as a self-reported measure [29, 34]. The presence of gingival bleeding in individuals with periodontitis amplified indeed the magnitude of the association with MetS components, possibly expressing the composite effect of local and systemic inflammation [18]. In fact, among the hypotheses behind the association of MetS components with periodontal diseases is that of chronic low-grade inflammation, which may support their parallel development and worsening [14, 35]. Interestingly, the phenomenon of immunomodulation is at least in part sex-specific, and some evidence indicates that the female sex might be more susceptible to the association between MetS and periodontitis [14, 30]. Specifically, a recent small intervention study in a cohort of individuals diagnosed with MetS found that exposure to a diet restricted by 300–500 kcal daily for two weeks was associated with a decrease in gingival bleeding, assessed by BoP, in women, but not in men [36]. Our findings support and expand this observation by identifying an association between MetS components and bleeding gums that is specific for women. This evidence opens the door to a new horizon of gender-based research and precision periodontology. From a mechanistic point of view, although the molecular events underlying the association remain unclear, some role for inflammatory mediators has been reported. For instance, hyperglycemia was associated with both high serum CRP levels and increased periodontal damage [37, 38]. In parallel, studies on rats found that periodontitis was aggravated by MetS and that interleukin-6 (IL-6) and interleukin-1 (IL-1) mediated osteoclasts activation [39]. Hypertriglyceridemia also plays an important role in periodontitis [39]. In particular, high concentrations of fatty acids (palmitic acid) were found to amplify the expression of IL-1 alpha, IL-1 beta, CXCL10, CD14, CD86, CSFs-2, MCP-1, TLRs, and TNF-α [40]. In support of this, in vitro studies on macrophages demonstrated an upregulation of CD36 + cells, involved in adipogenesis and triglycerides accumulation, after treatment with a combination of lipopolysaccharide and palmitic acid compared to treatment with lipopolysaccharide alone or palmitic acid alone [40]. Elevated CD36 counts were independently associated with periodontitis and MetS [40], especially if occurring together, and CD36 expression in periodontal tissues was positively correlated with osteoclastogenesis [40]. In turn, dysbiotic shifts in the oral and the gut microbiome composition could represent another potential mechanism behind the association between periodontal diseases and MetS [41, 42]. Indeed, the salivary burden of 4 major periodontal bacteria was found to be independently associated with low HDL-cholesterol and high triglycerides levels [43]. Mechanistically, gut dysbiosis and the resulting aberrant metabolite concentrations in the gut lumen are thought to weaken tight junction integrity, thereby allowing bacterial products to trespass the epithelial barrier and enter the circulation [41]. Through the bloodstream, such products reach the liver and adipose tissue, interact with hepatocytes, adipocytes, and local immune cells, and elicit the production of proinflammatory cytokines and chemokines that contribute to the chronic low-grade inflammation and insulin resistance typical of MetS [41, 44, 45].

Interestingly, we found that the only MetS factor associated with periodontal diseases in men was high BP. This expands with sex-specific data the already growing evidence of an independent association between periodontal diseases and arterial hypertension [46] and poor BP control [19], especially in the presence of gingival bleeding [18], possibly supported by multiple and sexually dimorphic immunological and non-immunological mechanisms.

This study had limitations that need to be discussed. First, data might not be generalizable to non-US populations, and the lack of an external validation cohort should be considered when interpreting the results; second, since NHANES used a two-site per-tooth assessment method, a certain degree of underestimation of periodontitis prevalence should be considered; third, information on the use of hypoglycemic agents, antihypertensives medications, and smoking was self-reported. In addition, it should be considered that some variables with possible impact on the association, such as vitamin D levels, serum and urinary calcium, and degree of access to dental care, all of which may be relevant for the estimates, were not available to the present study [47]. However, this study also had strengths that deserve to be mentioned. It is based on the analysis of a large, multi-ethnic cohort where participants were uniformly paired through a robust allocation method that ensured sex, age, racial/ethnic, and income balance, while also allowing the homogeneous distribution of major confounders, like smoking. Sex matching allowed to conduct balanced sex-specific analyses that expand on current knowledge in the field of gender periodontology. Rigorous statistical techniques were used to challenge the results. A technology as innovative as ML was used to challenge the predictive power of the MetS component in terms of periodontal health status.

In conclusion, this nested cross-sectional study with paired groups confirms a certain degree of association between MetS components and periodontal diseases and adds to the available literature with women-specific data on the independent association of Mets with gingival bleeding. Mechanistic and intervention studies are needed to confirm these observations in similar and different settings before they can be transferred into clinical practice.

## Supplementary Information

**Additional file 1: Figure S1.** Data reduction diagram. **Figure S2.** Risk of bleeding and non-bleeding periodontal diseases based on the number of MetS components. Overall results are presented. **Table S1.** Sex-stratified demographic and clinical features of exposed and non-exposed NHANESIII individuals included in the study. **Table S2.** Results of RERI stratified by sex.

## Data Availability

Data can be download from cdc.gov website.

## Abbreviations

Mets: Metabolic syndrome
ML: Machine learning
BP: Blood pressure
HT: Hypertension
LDL-c: Low density lipoprotein cholesterol
HDL-c: High density lipoprotein cholesterol
WC: Waist circumference
CRP: C-reactive protein
IL: Interleukin
NHANES: National Health And Nutrition Examination Survey
CAL: Clinical attachment loss
BoP: Bleeding on probing
PPD: Periodontal probing depth
AAP: American Academy of Periodontology
EFP: European Federation of Periodontology
GBM: Gradient boosting model
RF: Random forest
XgbTree: EXtreme gradient boosting
AUCROC: Area under the receiver operating characteristics
HbA1c: Glycohemoglobin A1c
BMI: Body mass index
PISA: Periodontal inflamed surface area
WBC: White blood cells
