# Supplementary material for: Association between metabolic syndrome components and gingival bleeding is women-specific: a nested cross-sectional study
Source: J Transl Med. 2023 Apr 10;21:252. doi: 10.1186/s12967-023-04072-z (PMC10088168; doi:10.1186/s12967-023-04072-z)
Supplement: Supplementary file 1 — Additional file 1: Figure S1. Data reduction diagram. Figure S2. Risk of bleeding and non-bleeding periodontal diseases based on the number of MetS components. Overall results are presented. Table S1. Sex-stratified demographic and clinical features of exposed and non-exposed NHANESIII individuals included in the study. Table S2. Results of RERI stratified by sex. [file 12967_2023_4072_MOESM1_ESM.docx]

**ADDITIONAL FILE**

**Association between metabolic syndrome components and gingival bleeding is women-specific: a nested cross-sectional study**

*Pietropaoli D, Altamura S, Ortu E, Guerrini L, Pizarro TT, Ferri C and Del Pinto R.*

**S Figure 1**. Data reduction diagram.


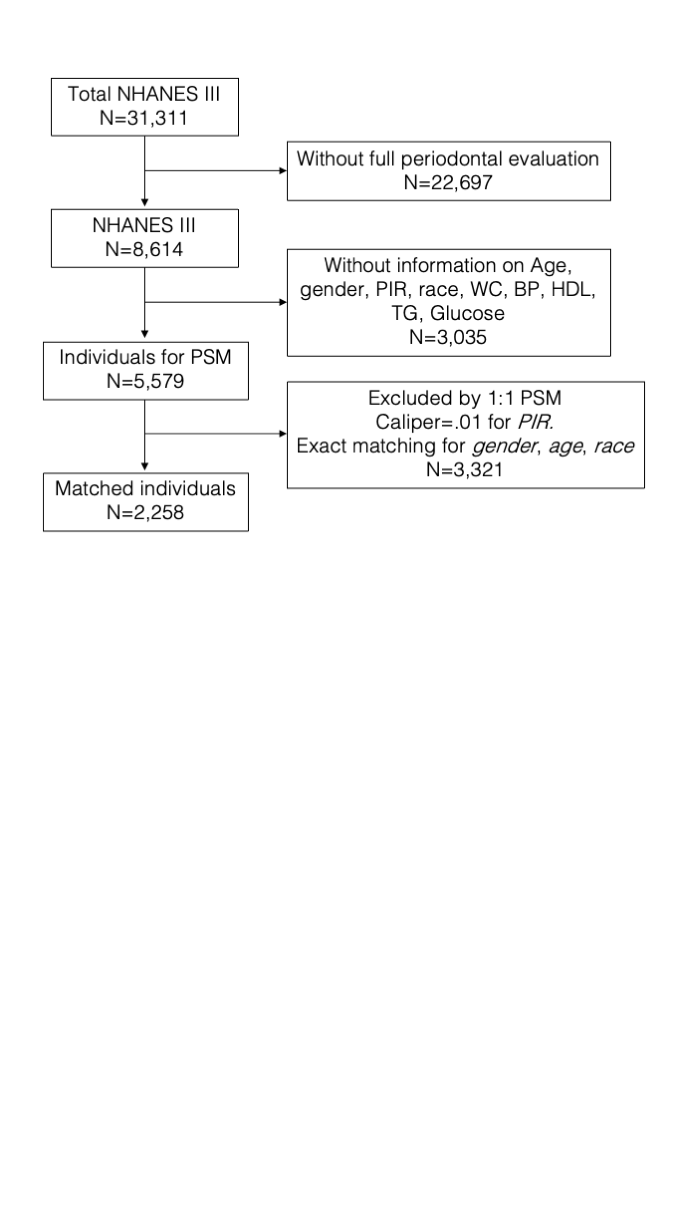


**S Figure 2: Risk of bleeding and non-bleeding periodontal diseases based on the number of MetS components.** Overall results are presented.


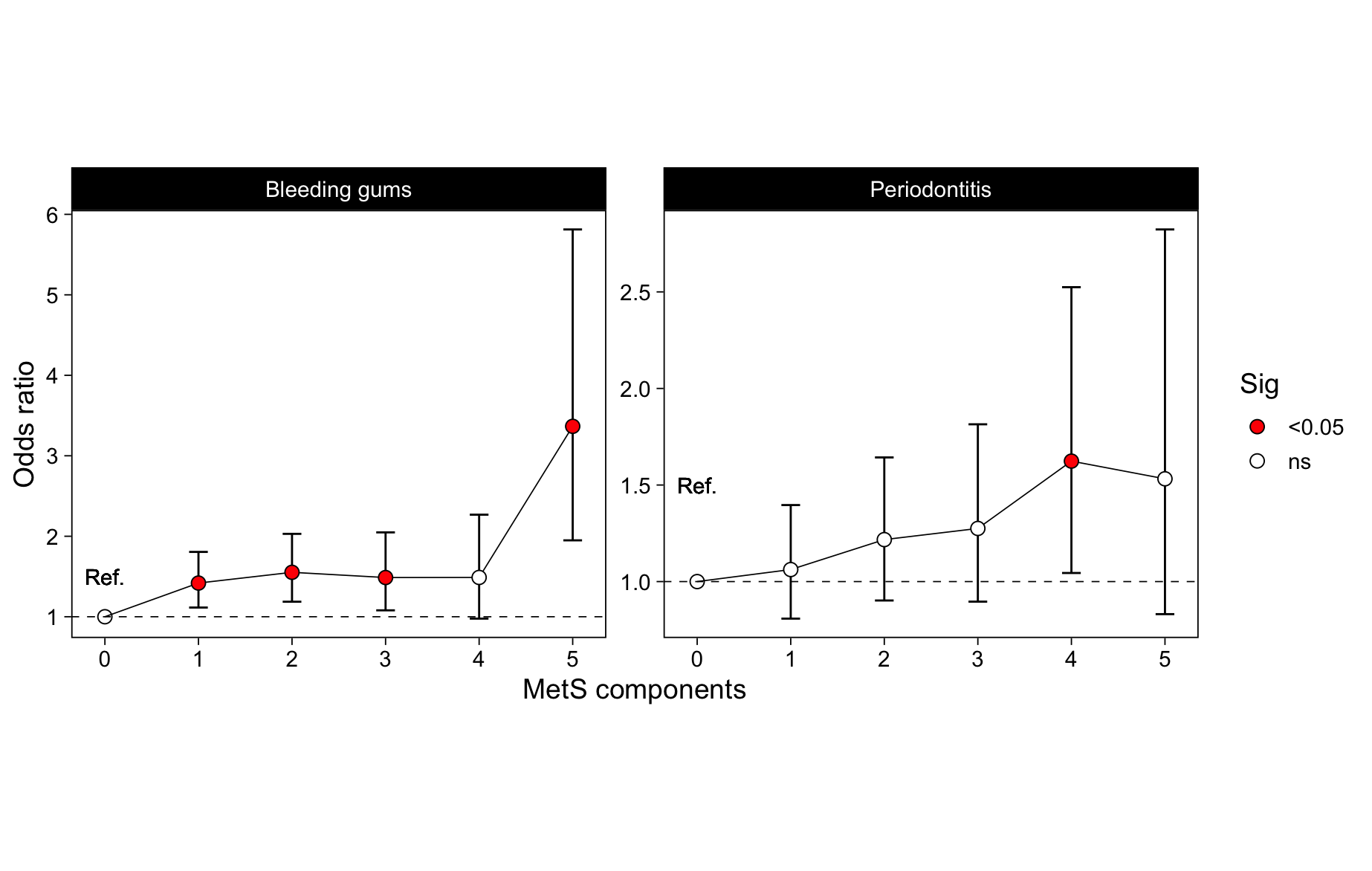


**S Table 1. Sex-stratified demographic and clinical features of exposed and non-exposed NHANESIII individuals included in the study.**

|  | | **Non-exposed to MetS components** | | **Exposed to at least 1 MetS component** | |  |
| --- | --- | --- | --- | --- | --- | --- |
|  | level | Male | Female | Male | Female | p |
| n |  | 555 | 574 | 555 | 574 |  |
| Periodontitis (%) |  | 160 (28.8) | 62 (10.8) | 164 (29.5) | 95 (16.6) | <0.001 |
| Periodontal status (%) | Healthy | 276 (49.7) | 429 (74.7) | 273 (49.2) | 337 (58.7) | <0.001 |
|  | Gingivitis | 119 (21.4) | 83 (14.5) | 118 (21.3) | 142 (24.7) |  |
|  | Stable periodontitis | 99 (17.8) | 44 (7.7) | 87 (15.7) | 50 (8.7) |  |
|  | Unstable periodontitis | 61 (11.0) | 18 (3.1) | 77 (13.9) | 45 (7.8) |  |
| Age, years (%) | <45 | 373 (67.2) | 410 (71.4) | 373 (67.2) | 410 (71.4) | 0.548 |
|  | 45-65 | 129 (23.2) | 119 (20.7) | 129 (23.2) | 119 (20.7) |  |
|  | >65 | 53 (9.5) | 45 (7.8) | 53 (9.5) | 45 (7.8) |  |
| Smokers (%) |  | 331 (59.6) | 230 (40.1) | 363 (65.4) | 233 (40.6) | <0.001 |
| PISA (mean (SD)) |  | 42.71 (75.24) | 18.43 (40.13) | 49.92 (91.25) | 36.64 (66.34) | <0.001 |
| PPD (mean (SD)) |  | 2.49 (0.50) | 2.22 (0.36) | 2.54 (0.54) | 2.31 (0.44) | <0.001 |
| CAL (mean (SD)) |  | 2.26 (1.07) | 1.81 (0.66) | 2.28 (1.04) | 1.89 (0.69) | <0.001 |
| BoP (mean (SD)) |  | 9.93 (14.91) | 5.04 (9.28) | 11.00 (16.18) | 9.28 (13.86) | <0.001 |
| Gums bleeding (%) | No BoP | 248 (44.7) | 348 (60.6) | 233 (42.0) | 284 (49.5) | <0.001 |
|  | BoP <10% | 127 (22.9) | 125 (21.8) | 127 (22.9) | 103 (17.9) |  |
|  | BoP ≥10% | 180 (32.4) | 101 (17.6) | 195 (35.1) | 187 (32.6) |  |

**S Table 2**. **Results of RERI stratified by sex**.

| MALE | | | |  | FEMALE | | | |
| --- | --- | --- | --- | --- | --- | --- | --- | --- |
| * | Smoke absent | Smoke present | Effect of Smoke within the strata of ExposedToMetsFactors |  | * | Smoke absent | Smoke present | Effect of Smoke within the strata of ExposedToMetsFactors |
|  | OR [95% CI] | OR [95% CI] | OR [95% CI] |  |  | OR [95% CI] | OR [95% CI] | OR [95% CI] |
| Non exposed to any MetS factor | 1 [Reference] | 2.21 [1.48, 3.29] | 2.21 [1.48, 3.29] |  | Non exposed to any MetS factor | 1 [Reference] | 1.36 [0.8, 2.31] | 1.36 [0.8, 2.31] |
| Exposed to alìt least 1 MetS factor | 0.82 [0.49, 1.35] | 2.34 [1.58, 3.46] | 2.86 [1.85, 4.42] |  | Exposed to at least 1 MetS factor | 1.58 [0.99, 2.53] | 2.32 [1.43, 3.76] | 1.47 [0.94, 2.28] |
| Effect of ExposedToMetsFactors within the strata of Smoke | 0.82 [0.49, 1.35] | 1.06 [0.78, 1.45] |  |  | Effect of ExposedToMetsFactors within the strata of Smoke | 1.58 [0.99, 2.53] | 1.7 [1.03, 2.83] |  |
| Multiplicative scale | 1.29 [0.72, 2.34] |  |  |  | Multiplicative scale | 1.08 [0.54, 2.15] |  |  |
| RERI | 0.31 [-0.73, 1.1] |  |  |  | RERI | 0.38 [-0.93, 1.51] |  |  |
| AP | 0.13 [-0.28, 0.43] |  |  |  | AP | 0.16 [-0.42, 0.51] |  |  |
| SI | 1.3 [0.59, 2.88] |  |  |  | SI | 1.4 [0.48, 4.07] |  |  |
